# Regulation of endocytic trafficking of transferrin receptor by optineurin and its impairment by a glaucoma-associated mutant

**DOI:** 10.1186/1471-2121-11-4

**Published:** 2010-01-19

**Authors:** Ananthamurthy Nagabhushana, Madhavi L Chalasani, Nishant Jain, Vegesna Radha, Nandini Rangaraj, Dorairajan Balasubramanian, Ghanshyam Swarup

**Affiliations:** 1 Centre for Cellular and Molecular Biology, Uppal Road, Hyderabad - 500 007, India; 2 L.V. Prasad Eye Institute, Hyderabad - 500 034, India

## Abstract

**Background:**

Optineurin is a multifunctional protein involved in several functions such as vesicular trafficking from the Golgi to the plasma membrane, NF-κB regulation, signal transduction and gene expression. Mutations in optineurin are associated with glaucoma, a neurodegenerative eye disease that causes blindness. Genetic evidence suggests that the E50K (Glu50Lys) is a dominant disease-causing mutation of optineurin. However, functional alterations caused by mutations in optineurin are not known. Here, we have analyzed the role of optineurin in endocytic recycling and the effect of E50K mutant on this process.

**Results:**

We show that the knockdown of optineurin impairs trafficking of transferrin receptor to the juxtanuclear region. A point mutation (D474N) in the ubiquitin-binding domain abrogates localization of optineurin to the recycling endosomes and interaction with transferrin receptor. The function of ubiquitin-binding domain of optineurin is also needed for trafficking of transferrin to the juxtanuclear region. A disease causing mutation, E50K, impairs endocytic recycling of transferrin receptor as shown by enlarged recycling endosomes, slower dynamics of E50K vesicles and decreased transferrin uptake by the E50K-expressing cells. This impaired trafficking by the E50K mutant requires the function of its ubiquitin-binding domain. Compared to wild type optineurin, the E50K optineurin shows enhanced interaction and colocalization with transferrin receptor and Rab8. The velocity of Rab8 vesicles is reduced by co-expression of the E50K mutant. These results suggest that the E50K mutant affects Rab8-mediated transferrin receptor trafficking.

**Conclusions:**

Our results suggest that optineurin regulates endocytic trafficking of transferrin receptor to the juxtanuclear region. The E50K mutant impairs trafficking at the recycling endosomes due to altered interactions with Rab8 and transferrin receptor. These results also have implications for the pathogenesis of glaucoma caused by the E50K mutation because endocytic recycling is vital for maintaining homeostasis.

## Background

**Figure 1:**
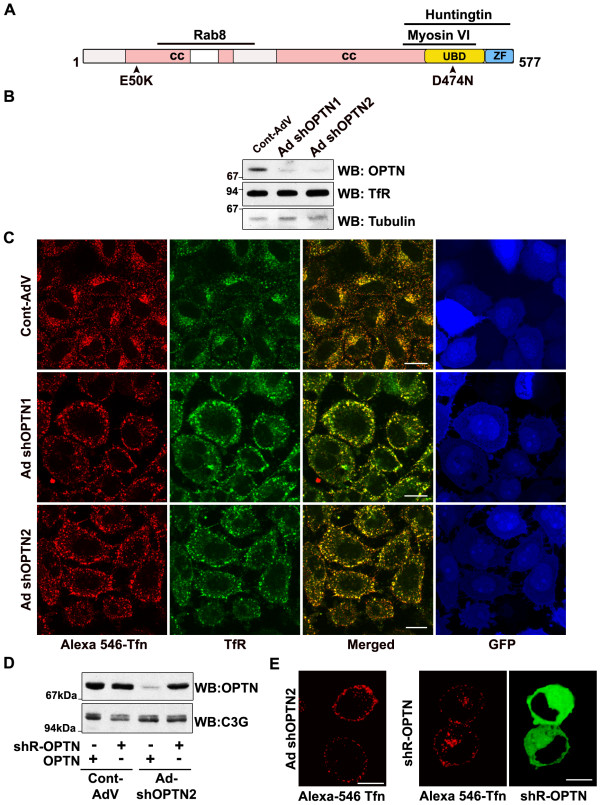
**Optineurin is required for the trafficking of TfR**. (A) Schematic representation of optineurin showing domain organisation, key mutations and binding sites of interacting proteins. UBD- Ubiquitin Binding Domain (424-511aa), CC-coiled coil domain, ZF- zinc finger domain. (B) HeLa cells were infected with adenoviruses expressing shRNAs (Ad shOPTN1 and Ad shOPTN2) against optineurin for 72 hrs. Western blots show levels of endogenous optineurin, TfR and tubulin. (C) HeLa cells were infected with adenoviruses expressing shRNAs for 72 hrs, serum starved for 2 hrs and then incubated with Alexa-546 conjugated transferrin for 15 min, stained for transferrin receptor and examined by confocal microscopy. (D) Expression of shRNA resistant mutant of optineurin in HeLa cells. (E) HeLa cells infected with adenoviruses expressing shRNA (Ad shOPTN2) were transfected with a shRNA resistant mutant of optineurin and were incubated with Alexa 546-Tfn for 15 min. Expression of shRNA resistant mutant (right panels, shown in green) rescues the effect of shRNA. Bar; 10 μm.

Optineurin is a multifunctional protein involved in a variety of functions such as vesicular trafficking from the Golgi to the plasma membrane, Golgi ribbon formation, signaling by metabotropic glutamate receptor, regulation of NF-κB activation and gene expression [1-6]. It is a 74 kDa protein that contains multiple coiled-coil domains, a leucine zipper, a ubiquitin-binding domain (UBD) and a C2H2 type zinc finger at its C-terminus (Figure 1A). Although optineurin is ubiquitously expressed, it shows high level of expression in certain tissues such as retina, brain, heart, skeletal muscle, placenta, testis and kidney [7-10]. Studies in various cell lines have shown that endogenous optineurin is present in the cytoplasm, Golgi and recycling endosome (RE) [1,11-14]. However, overexpressed optineurin is present more prominently in the RE [15].

Some of the optineurin- interacting proteins such as Rab8, huntingtin and myosinVI are involved in vesicular trafficking [1,14,16]. Rab8 is a member of family of small GTPases that regulate intracellular membrane trafficking pathways [17,18]. It is localized to vesicles at the trans-Golgi network (TGN), RE and membrane ruffles [16,19-21]. It plays an important role in membrane trafficking from the TGN to the plasma membrane in polarized epithelial and neuronal cells, and in membrane trafficking at the RE [19-24]. Huntingtin, a protein mutated in the neurodegenerative Huntington's disease, is localized to the Golgi complex, and to the endocytic and exocytic vesicles, where it plays a role in membrane trafficking pathways [14,25,26]. Myosin VI, an actin based motor protein, is found in diverse cellular compartments including the Golgi complex, membrane ruffles, endocytic vesicles and secretory vesicles [1,13]. Optineurin forms a bridge between myosin VI and Rab8 during sorting of cargo molecules in polarized epithelial cells [1,13]. Knockdown of optineurin affects Golgi ribbon formation and post-Golgi membrane trafficking to the plasma membrane [1]. However, the role of optineurin in endocytic membrane traffic has not been examined.

Endocytic membrane traffic is essential for the delivery of various membrane components, receptors and their ligands to their respective intracellular compartments [27]. Transferrin and transferrin receptor (TfR) regulate iron uptake in almost all cell types, and are frequently used to study endocytosis and recycling. The iron-bound transferrin is endocytosed through TfR and delivered to the peripheral early/sorting endosome [27]. Most of the internalized TfR is recycled back either through a fast, direct step from the early endosome, typically with a t_1/2 _of 2-3 min, or a slower step via the recycling endosome, with a t_1/2 _of ~ 10-15 min. The RE is a network of tubular structures that is perinuclear or juxtanuclear in most cells [28-30]. It is a relatively long lived compartment and acts as a sorting station for the endocytic and exocytic cargo [27]. The transport of cargo like TfR from the RE to plasma membrane is regulated by Rab11, whereas Rab8 regulates endocytic traffic to the RE [21,31-33]. As transport through RE is a relatively slow process, at steady state most of the receptors, like TfR, that are recycled, are localized mostly at the endocytic recycling compartment [34].

Mutations in optineurin, encoded by the *OPTN *gene, are associated with certain glaucomas, a group of neurodegenerative eye diseases that cause blindness [11,35]. However, functional alterations caused by mutations in optineurin are not known. Genetic evidence suggests that the E50K (Glu50Lys) is a dominant disease-causing mutation of optineurin which was found in 13.5% of families with hereditary glaucoma [11]. Previously we have analyzed the effect of overexpression of the E50K and other mutants on survival of various cells [36]. During this work we observed that, as compared to wild type optineurin, the E50K mutant forms large vesicle-like structures in many cells. This observation raised the possibility that the formation of large vesicle-like structures by the E50K mutant could be due to impaired trafficking. Here, we have analyzed the role of optineurin in endocytic recycling and the effect of E50K mutant on this process. Our results show that knockdown of optineurin results in slower trafficking of TfR to the juxtanuclear region. We show that optineurin interacts with TfR and inactivation of the UBD of optineurin by a point mutation abrogates this interaction. The results of several experiments suggest that the E50K mutant causes impaired trafficking at the RE due to enhanced interaction of this mutant with Rab8 and TfR. The UBD of optineurin plays an essential role in its localization to the RE and also in impaired trafficking by the E50K mutant.

## Results

### Optineurin is required for trafficking of transferrin receptor

We examined the role of optineurin in trafficking of TfR, which has served as a model system for the study of endocytic recycling. For this purpose, two shRNAs (short hairpin RNAs) which target two different regions of optineurin mRNA were used to knockdown endogenous optineurin. Infection of HeLa cells with the adenoviruses expressing shRNAs (Ad-shOptn1 and Ad-shOptn2) resulted in 70-80% decrease in optineurin protein level, as determined by western blotting (Figure 1B). The level of TfR was unaffected by these shRNAs (Figure 1B). An adenovirus expressing shRNA of unrelated sequence of the same length was used as a control. HeLa cells were infected with adenoviruses for 72 hours, serum starved for 2 hours and then incubated with Alexa546-labeled transferrin. After 15 minutes labeled transferrin was seen concentrated in the juxtanuclear region (corresponding to recycling endosomes) in control cells. Upon knockdown of endogenous optineurin by two different shRNAs, most of the internalized transferrin was found distributed throughout the cell especially in the cell periphery (Figure 1C). The distribution of endogenous TfR was similar to that of the labeled transferrin (Figure 1C). Upon chase with unlabeled transferrin for 30 -45 min, most of the transferrin was externalized both from the control and optineurin depleted cells (data not shown). To rule out the possibility of non-specific effects of shRNA, we generated an shRNA resistant mutant of optineurin (Figure 1D). Expression of the shRNA-resistant mutant in knockdown cells restored the distribution of labeled transferrin to the juxtanuclear region (Figure 1E). These results suggest that optineurin is required for the trafficking of TfR to the juxtanuclear region. These results also indicate that in optineurin knockdown cells the sorting endosomes are taking up most of the TfR or the recycling endosome is disrupted spatially or functionally, or both.

### The UBD is required for localization of optineurin to the recycling endosomes

**Figure 2:**
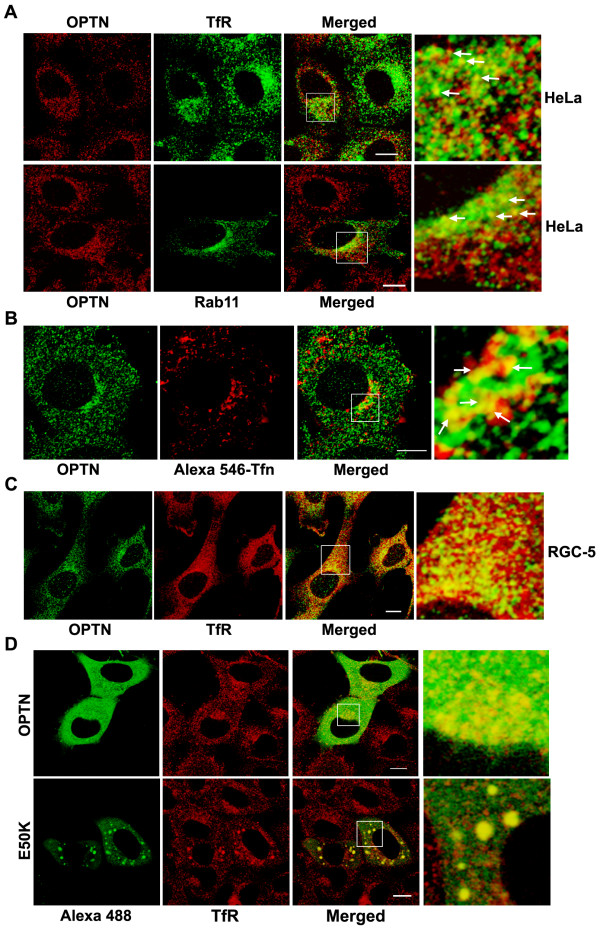
**Optineurin is localized to the recycling endosomes**. (A) Endogenous optineurin is localized to recycling endosomes in HeLa cells. HeLa cells were stained sequentially for endogenous optineurin and TfR (upper panel) or were transfected with GFP-Rab11 and stained for endogenous optineurin (lower panel). Arrows indicate vesicles showing co-localization. (B) HeLa cells were pulsed with Alexa-546 conjugated transferrin for 30 min, chased with unlabeled holo-transferrin for 20 min and stained for endogenous optineurin. Arrows indicate vesicles showing colocalization. (C) RGC-5 cells were stained for endogenous optineurin and TfR. (D) E50K mutant optineurin forms large vesicles and co-localizes with TfR. HeLa cells were transfected with either E50K mutant or wild-type optineurin expressing plasmids lacking a tag and stained sequentially for optineurin and TfR. E50K mutant optineurin forms large vesicular structures that co-localize strongly with TfR indicated by yellow colour in the merged image. Right panels show enlarged image of boxed area in merged panels. Bar; 10 μm.

**Figure 3:**
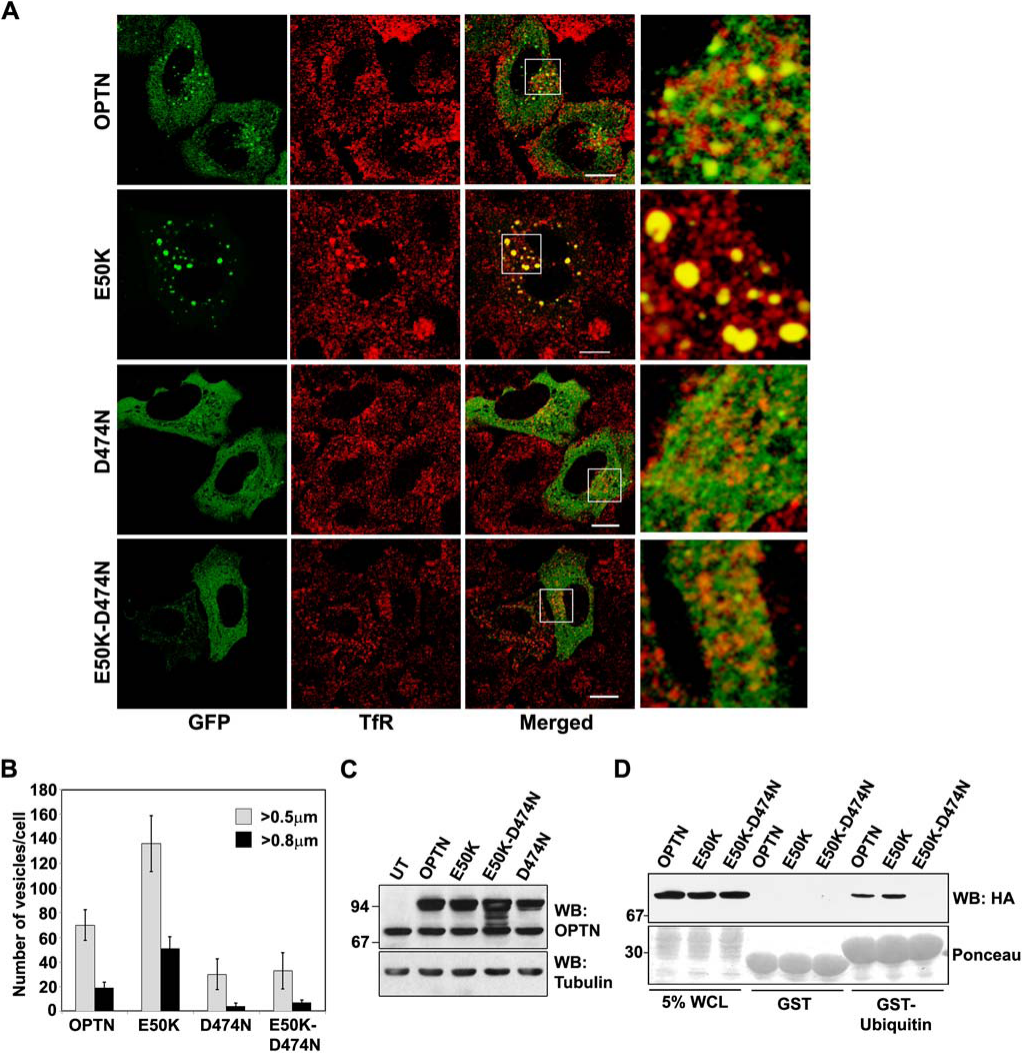
**The UBD is required for localization of optineurin to RE**. (A) HeLa cells were transfected with GFP-tagged wild type optineurin or its mutants and stained for TfR. Ubiquitin binding defective D474N and E50K-D474N mutants are not recruited to RE as indicated by a lack of colocalization with TfR. Note the absence of large vesicular structures in E50K-D474N mutant. Bar; 10 μm. (B) The number of vesicles in cells expressing GFP tagged wild-type and mutant optineurin. (C) Western blot showing expression of GFP tagged optineurin and its mutants, probed with optineurin antibody. (D) E50K-D474N mutant does not bind ubiquitin. GST-ubiquitin or GST alone bound to glutathione agarose beads were incubated with lysates of HeLa cells transfected with wild type optineurin or its mutants. The bound proteins were eluted and immunoblotted with anti-HA antibodies. WCL, 5% whole cell lysate.

**Figure 4:**
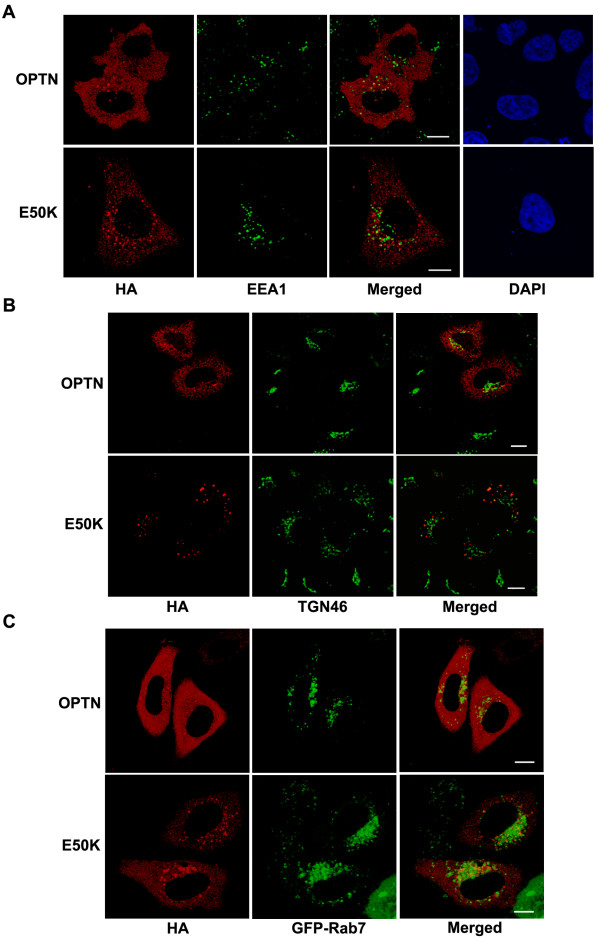
**Characterization of subcellular localization of optineurin and E50K mutant**. HeLa cells were transfected with HA-tagged wild type or E50K mutant optineurin and stained for (A) an early endosome marker EEA1 or (B) TGN46, a marker for trans-Golgi or (C) co-transfected with GFP-Rab7, a marker for late endosome.

**Figure 5:**
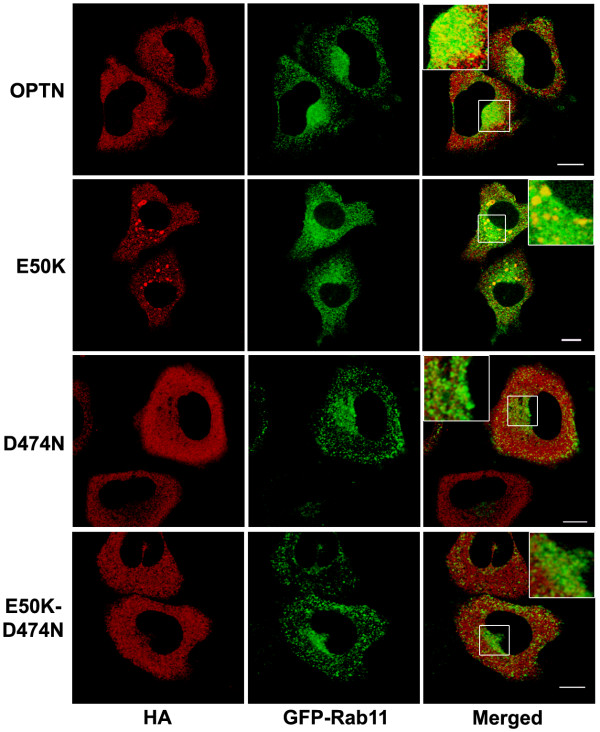
**Colocalization of optineurin and E50K mutant with Rab11**. HeLa cells were co-transfected with HA-tagged wildtype optineurin or its mutants and GFP-Rab11. Both wildtype optineurin and E50K mutant show colocalization with Rab11. Insets show enlarged region of the boxed area. Correlation coefficients for co-localization, wild-type optineurin, 0.32 ± 0.04; E50K, 0.45 ± 0.08; D474N, 0.07 ± 0.03; E50K-D474N, 0.08 ± 0.04.

We examined the localization of endogenous optineurin in HeLa cells and found that it showed some amount of colocalization with TfR and Rab11 in some of the vesicles present in the juxtanuclear region (Figure 2A). Both TfR and Rab11 are markers of RE [27-31,37]. To further validate these observations, HeLa cells were pulsed with labeled transferrin for 30 min, chased with unlabelled transferrin for 20 min and stained for endogenous optineurin. A fraction of endogenous optineurin showed colocalization with transferrin (Figure 2B). Similarly endogenous optineurin showed some colocalization with TfR in RGC-5 cells, a retinal ganglion cell line (Figure 2C). These results showed that at least a small fraction of endogenous optineurin is present in the recycling endosomes; this is in addition to its presence in the Golgi reported previously. Overexpressed optineurin without tag and its E50K mutant also showed colocalization with TfR (Figure. 2D). Vesicles formed by the E50K mutant showed better colocalization with TfR compared to vesicles formed by wild type optineurin (correlation coefficient, E50K mutant 0.59 ± 0.11, wild type optineurin 0.29 ± 0.07; p < 0.05) (Figure 2D). The E50K mutant expressed without tag (Figure 2D) or with GFP tag (Figure 3A) formed larger vesicle-like structures than wild type optineurin mostly in the juxtanuclear region (Table 1). The cells expressing E50K mutant showed larger number of vesicle-like structures than those expressing wild type optineurin (Figure 3B). Formation of larger vesicle-like structures by GFP-E50K was not due to its higher level of expression as compared to GFP-optineurin (Figure 3C). We then analyzed colocalization of overexpressed optineurin and its E50K mutant with markers of early endosome (EEA1), late endosome (Rab7) and trans-Golgi network (TGN46). Overexpressed optineurin and the E50K mutant did not show any significant colocalization with EEA1, although few vesicles showed colocalization with Rab7 and TGN46 (Figure 4A-C). However, both E50K mutant and wild type optineurin showed significant colocalization with Rab11 (correlation coefficient, E50K mutant 0.45 ± 0.08, wild type optineurin 0.32 ± 0.04) (Figure 5). These results suggest that most of the E50K vesicles are not likely to be routed to late endosome or lysosome although a small fraction might be doing so.

**Table 1:** Sizes of vesicles formed by optineurin and its mutants

Vesicles formed by	Size in μm
Wild-type optineurin	0.79 ± 0.18
E50K	1.38 ± 0.43
D474N	0.51 ± 0.15
E50K-D474N	0.58 ± 0.16

A recent study showed the presence of a novel UBD in optineurin, and the D474N mutation in this UBD abolished the binding of optineurin to ubiquitinated proteins [3]. To explore the role of the UBD in the recruitment of E50K mutant to recycling endosomes, we generated the D474N mutant and a double mutant of optineurin that contains both E50K and D474N mutations (Figure 1A). The D474N mutant formed smaller and fewer vesicle-like structures than those formed by wild type optineurin (Table 1 and Figure 3B). The D474N mutant showed very little colocalization with TfR (correlation coefficient, D474N mutant 0.10 ± 0.03, wild type optineurin 0.38 ± 0.11) (Figure 3A) or Rab11 (Figure 5). The E50K-D474N double mutant also formed few small vesicle-like structures similar to D474N mutant, and did not show any significant colocalization with TfR (correlation coefficient, double mutant 0.08 ± 0.04, E50K mutant 0.58 ± 0.10) (Table 1; Figure 3A and 3B) or Rab11 (Figure 5). We then examined the ability of optineurin and E50K mutant to bind ubiquitin in an *in vitro *assay. Lysates of HeLa cells transfected with wild type optineurin or E50K or E50K-D474N mutants were incubated with GST-ubiquitin and bound proteins were analyzed by Western blot. As compared to wild type optineurin, E50K mutant showed a marginal increase in binding with GST-ubiquitin (Figure 3D). The E50K-D474N double mutant did not show any binding to GST-ubiquitin (Figure 3D). These results suggest that the UBD plays an essential role in the localization of optineurin and its E50K mutant to the RE and in the formation of large vesicle-like structures by E50K mutant.

### The UBD of optineurin is required for trafficking of transferrin to the juxtranuclear region

**Figure 6:**
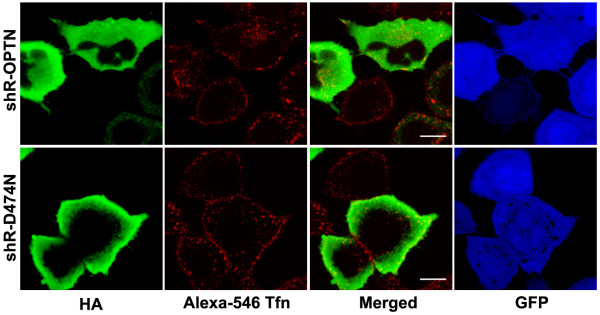
**The UBD is required for trafficking of transferrin**. HeLa cells infected with adenoviruses expressing shRNA (Ad-shOPTN2) were transfected with shRNA resistant wild type or D474N mutant optineurin. Cells were incubated with Alexa 546-Tfn for 15 min, fixed, stained for optineurin and examined by confocal microscopy.

Since UBD was required for localization of optineurin to the RE, we examined its role in trafficking of labeled transferrin to the RE. For this purpose we generated shRNA-resistant D474N mutant of optineurin. While re-introduction of shRNA resistant wild-type optineurin could restore distribution of transferrin to the juxtanuclear region, in cells expressing shRNA resistant D474N mutant, transferrin was found distributed throughout the cell (Figure 6). These observations suggest that a functional UBD is required for trafficking of transferrin to the juxtanuclear region.

### The E50K mutant causes reduced uptake of transferrin

**Figure 7:**
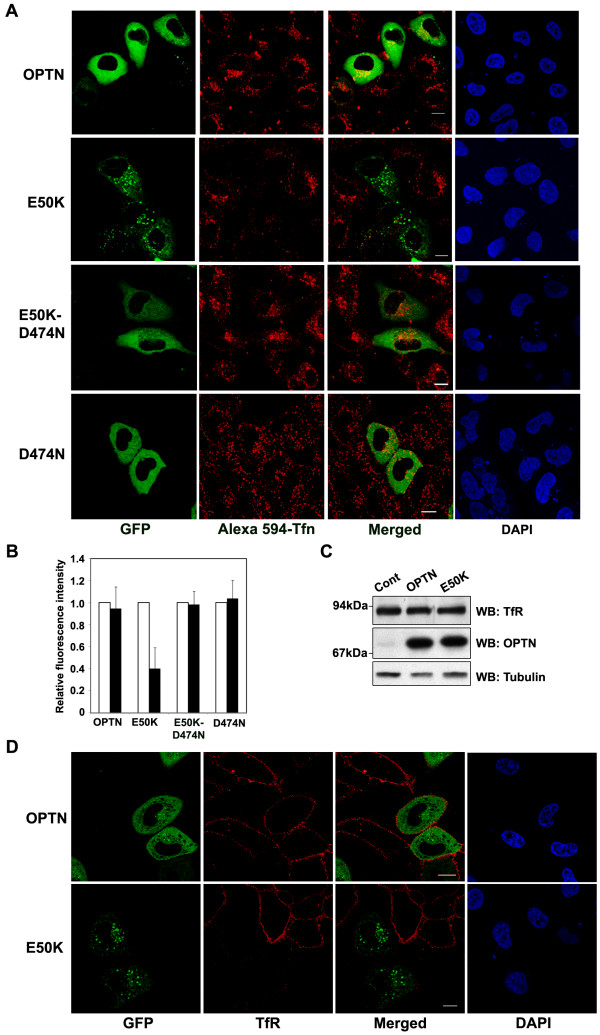
**The E50K mutant causes reduced uptake of transferrin**. (A) HeLa cells were transfected with GFP- tagged wild type optineurin or its mutants, serum starved and incubated with Alexa-594 conjugated transferrin for 20 min. Cells were then fixed and observed with a confocal microscope. (B) The uptake of labelled transferrin was quantitated by measuring the fluorescence intensity in expressing (filled bar) and non-expressing (empty bar) cells. (C) Western blot showing expression of TfR in cells infected with adenoviruses expressing wild-type or E50K mutant optineurin or control viruses. Western blot shows levels of TfR, optineurin and tubulin. (D) HeLa cells were transfected with GFP-tagged wild-type or E50K mutant optineurin. TfR present at the plasma membrane was visualized by staining these cells without permeablilzation using TfR antibody.

We examined the effect of over expressed optineurin and E50K mutant on trafficking of transferrin. HeLa cells transfected with either wild-type or E50K optineurin were incubated with Alexa594-conjugated transferrin. To our surprise the uptake of transferrin was significantly reduced in majority of the cells expressing mutant optineurin (Figure 7A). Quantitative analysis showed that there was 60% inhibition (P < 0.001) of uptake of labeled transferrin by mutant optineurin, whereas wild type optineurin showed no significant inhibition (Figure. 7B). The reduction in uptake of transferrin was not due to decreased level of total cellular TfR in E50K expressing cells (Figure 7C). However, the amount of cell surface associated TfR in the E50K expressing cells was decreased (Figure 7D), suggesting that the reduced uptake of transferrin by the E50K expressing cells was due to a reduction in TfR on the cell surface. The expression of either D474N mutant or double mutant E50K-D474N did not cause any reduction in the uptake of labeled transferrin (Figure 7A, B), indicating therefore that the interactions of UBD are required for the effect of E50K on transferrin uptake. Neither the steady state localization of TfR nor trafficking of transferrin was affected by overexpression of D474N or E50K-D474N mutant suggesting that these mutants possibly do not affect the function of endogenous optineurin. The formation of transferrin receptor-positive large vesicles by the E50K mutant and the decreased uptake of transferrin, suggest that the E50K optineurin causes a reduction in endocytic recycling of transferrin receptor, leading to its accumulation in the large vesicular structures.

### The E50K mutant exhibits slower dynamics

**Figure 8:**
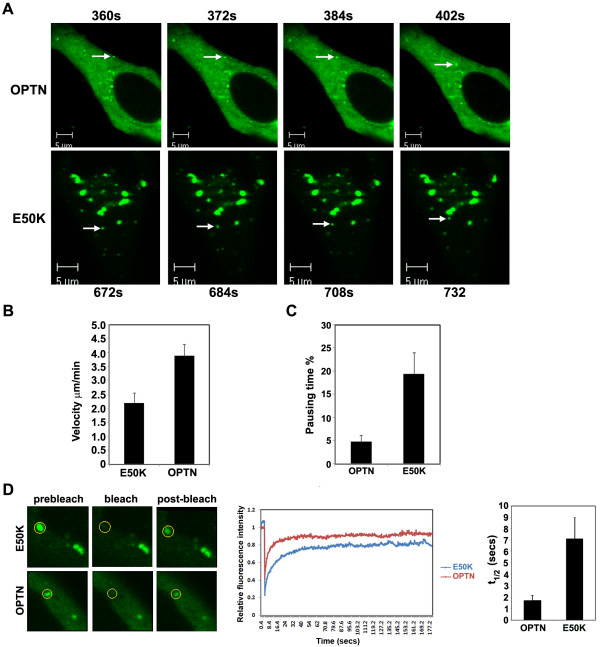
**The E50K mutant exhibits slower dynamics**. (A) Dynamics of optineurin and E50K mutant vesicles. Individual frames from a movie of cells transfected with GFP-optineurin (upper panels) or GFP-E50K (lower panels) with the images acquired every 12 secs. Frame sequence illustrates the movement of a vesicle (denoted by an arrow). Additional files 1 and 2 show the movement of these vesicles. Bar; 5 μm. (B) Velocities of wild type and E50K mutant optineurin vesicles. E50K vesicles move significantly slower compared to wild type optineurin. (C) Pausing time of GFP-optineurin and GFP-E50K vesicles. (D) HeLa cells were transfected with GFP-tagged E50K or wild type optineurin. A representative fluorescence recovery curve after photobleaching is shown. Right panel shows t_1/2 _time for E50K and optineurin.

The dynamics of the optineurin-containing vesicles was analyzed using time-lapse video microscopy. GFP-tagged wild type or E50K optineurin cDNAs were transfected into HeLa cells and the vesicle movement monitored by acquiring a series of time lapse images (Figure 8A and Additional files 1 and 2). A large number of these vesicles moved inwards from the cell periphery towards the nucleus/Golgi body (though a few moved outward). Occasional fusing of the smaller vesicles with larger ones was observed, mainly in the perinuclear region. Analysis of velocities of vesicles of largely comparable sizes revealed that wild type optineurin vesicles moved at an average velocity of 3.9 ± 0.4 μm/min, while those containing the mutant moved significantly slower (2.2 ± 0.36 μm/min) (p < 0.001) (Figure 8B). The E50K-D474N containing vesicles moved at an average velocity of 4.72 ± 0.42 μm/min. We then determined the pausing of vesicles by measuring the time these vesicles spend without moving and found that E50K vesicles paused for a longer period of time compared with wild type optineurin vesicles (Figure 8C).

Fluorescence recovery after photo bleaching (FRAP) is a widely used method to measure two dimensional diffusion of molecules in live cells. We further analyzed the dynamics of E50K mutant and wild-type optineurin in the vesicles of comparable size by using FRAP. These experiments showed that the half time of recovery of fluorescence in the E50K-containing vesicles was higher (t 1/2 = 7.13 ± 1.865 secs) than those with the wild type molecule (t 1/2 = 1.68 ± 0.489 secs; P < 0.01) (Figure 8D).

### Optineurin interacts with transferrin receptor through UBD

**Figure 9:**
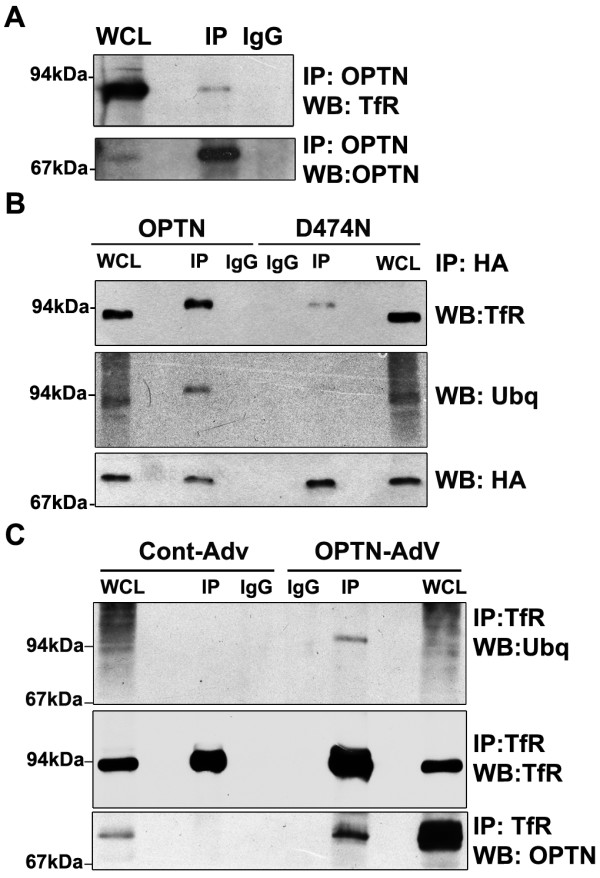
**Optineurin interacts with TfR through UBD**. (A) Endogenous optineurin interacts with endogenous TfR. Endogenous optineurin was immunoprecipitated from HeLa cells using anti-optineurin or control antibody. Immunoprecipitates were resolved in SDS-PAGE and subjected to Western blotting with TfR and optineurin antibodies. (B) UBD of optineurin is essential for interaction with TfR. HeLa cells were transfected with HA-optineurin or HA-D474N mutant optineurin. After 30 hrs of transfection, lysates were immunoprecipitated with anti-HA or control antibody and subjected to Western blotting with TfR, ubiquitin and HA antibodies. (C) TfR was immunoprecipitated from optineurin-overexpressing and control cells. Western blot of the immunoprecipitate was done with ubiquitin, TfR and optineurin antibodies. WCL, 5% whole cell lysate.

The results so far showed that optineurin is required for TfR trafficking and it is localized to the recycling endosomes through its UBD. How does the UBD help in recruitment or retention of optineurin in the recycling endosomes? One possibility is that optineurin may form a complex with a ubiquitinated protein present in the recycling endosomes. Since optineurin colocalizes with TfR very well, we examined the possibility of complex formation between optineurin and TfR. To analyze the interaction between endogenous optineurin and TfR, HeLa cell lysates were immunoprecipitated with optineurin antibody. TfR was detected in the immunoprecipitate with optineurin antibody but not with control antibody (Figure 9A). To examine the possible role of the UBD of optineurin in complex formation with TfR, we used the D474N mutant which does not bind ubiquitin. HeLa cells were transfected with either HA-tagged wild-type or D474N mutant optineurin and immunoprecipitation was carried out with HA antibody. While TfR was detected in the immunoprecipitate from wild type optineurin expressing cells, inactivation of the UBD resulted in 80% reduction of complex formation between optineurin and TfR (Figure 9B). Since the interaction between optineurin and TfR was dependent on intact UBD, it raised an interesting possibility that TfR complexed with optineurin could be ubiquitinated. Reprobing of the blot with ubiquitin antibody suggested that TfR, that was complexed with overexpressed optineurin, was ubiquitinated (Figure 9B). To provide further evidence for ubiquitination of TfR, we immunoprecipitated it from optineurin-overexpressing and control cells. Western blot of the immunoprecipitate with ubiquitin antibody showed a band moving slower than the bulk of the immunoprecipitated TfR, comigrating with the upper band of TfR in optineurin-overexpressing cells (Figure 9C). In the control cells we could not detect ubiquitinated TfR. These results suggest that ubiquitination of TfR is transient and overexpression of optineurin enhances this ubiquitination.

### The E50K mutant shows enhanced interaction with transferrin receptor

**Figure 10:**
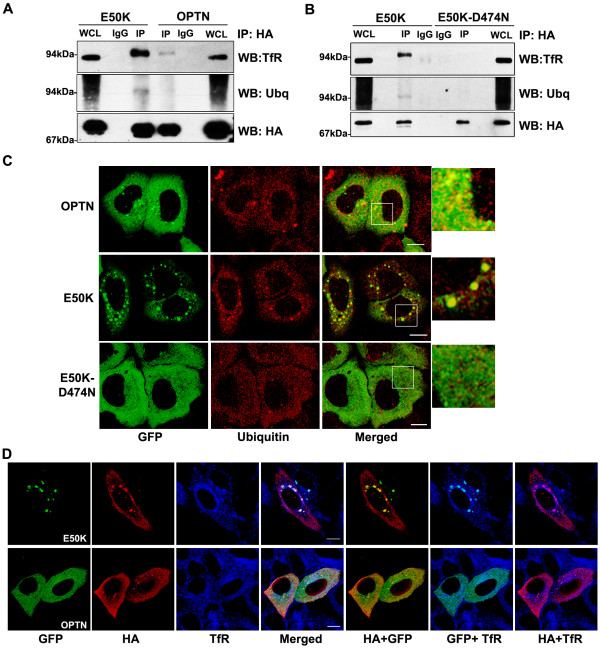
**The E50K mutant shows enhanced interaction with TfR**. (A) E50K mutant shows enhanced interaction with TfR. HeLa cells were transfected with HA-tagged E50K mutant or wild type optineurin. After 30 hrs of transfection, lysates were prepared and immunoprecipitated with HA or control antibody and subjected to Western blotting with TfR, ubiquitin and HA antibodies. WCL, 5% whole cell lysate. (B) Interaction of E50K mutant optineurin with TfR is mediated through UBD. HeLa cells were transfected with HA-tagged E50K mutant or E50K-D474N mutant optineurin and immunoprecipitation was carried out as described in (A). WCL, 5% whole cell lysate. (C) E50K mutant colocalizes with ubiquitin. HeLa cells were transfected with GFP-tagged constructs of wild-type optineurin or its mutants and stained for endogenous ubiquitin. (D) E50K mutant colocalizes with ubiquitin in recycling endosomes. HeLa cells were co-transfected with GFP-tagged constructs of wild-type optineurin or E50K and HA-ubiquitin and stained with TfR and HA antibodies.

We compared the ability of optineurin and its E50K mutant to form complex with TfR by immunoprecipitation. In the E50K immunoprecipitate, 7 fold higher level of TfR was present compared to the optineurin immunoprecipitate, as determined by Western blot (Figure 10A). The role of the UBD in complex formation between E50K and TfR was examined by using E50K-D474N double mutant which showed a drastic reduction in complex formation with TfR (Figure 10B). Reprobing of this blot with ubiquitin antibody suggested that the TfR immunoprecipitated by E50K is ubiquitinated (Figure 10B). Vesicles formed by the E50K mutant and wild type optineurin showed colocalization with endogenous ubiquitin (Figure 10C). The double mutant E50K-D474N formed very few small vesicles which did not show significant colocalization with ubiquitin (Figure 10C). Staining for transferrin receptor in the cells co-transfected with HA-ubiquitin and wild-type or E50K mutant optineurin showed that ubiquitin colocalizes with both E50K mutant and transferrin receptor in same vesicular structures (Figure 10D). These results suggest that optineurin and the E50K mutant are recruited to the RE (or retained in the RE) by complex formation with a ubiquitinated protein, possibly TfR. These results also indicate that formation of larger vesicles (RE) by E50K might be due to formation of stronger complex with TfR.

### The E50K mutant is altered in its interaction with Rab8

**Figure 11:**
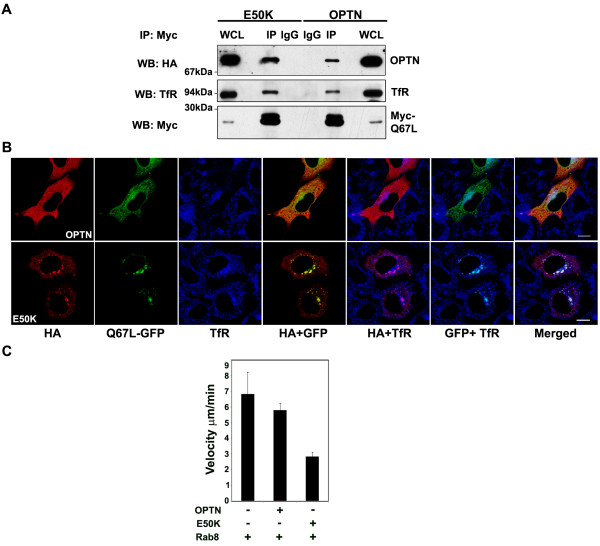
**The E50K mutant is altered in its interaction with Rab8**. (A) E50K mutant shows enhanced interaction with activated Rab8. HeLa cells were infected with adenoviruses expressing HA-E50K or HA-optineurin and then transfected with Myc-Q67L-Rab8 mutant. After 36 hrs of infection, lysates were prepared and immunoprecipitated with anti-Myc or control antibody and subjected to Western blotting with TfR, HA and anti-Myc antibodies. WCL, 5% whole cell lysate. (B) Co-localization of E50K mutant with Q67L-Rab8 and TfR. HeLa cells were cotransfected with GFP-tagged Q67L-Rab8 along with HA- tagged E50K mutant or wild type optineurin. Cells were stained sequentially with antibodies against HA-tag (red) and transferrin receptor (blue). Pairwise colocalizations are shown as indicated. Bar; 10 μm. (C) E50K mutant affects velocity of Rab8 vesicles. HeLa cells were co-transfected with HA-E50K or HA-optineurin and GFP-Rab8 and the movement of Rab8 vesicles was tracked to measure their velocities. In cells co-expressing E50K velocity of Rab8 vesicles is reduced compared to those expressing wild type optineurin or Rab8 alone.

Rab GTPases control various membrane trafficking pathways in the cell and Rab8 is involved in regulating exocytic and recycling membrane trafficking at the RE [20-24]. Optineurin interacts directly with the constitutively active GTP bound Q67L-Rab8 mutant but not inactive T22N mutant [16]. It is therefore believed that optineurin is an effector of some of the functions of Rab8. Rab8 is also required for the trafficking of TfR to the juxtanuclear region [21]. We next tested the hypothesis, that the E50K mutant of optineurin might be altered in its interaction with Rab8, by immunoprecipitation experiments. HeLa cells were cotransfected with Myc-tagged Q67L-Rab8 mutant and wild-type or E50K mutant optineurin. Immunoprecipitation experiments revealed that in the Rab8 immunoprecipitate 2.9 fold higher amount of E50K was present compared to wild-type optineurin (Figure 11A). This suggests that as compared with optineurin, E50K forms a stronger complex with Q67L-Rab8. Transferrin receptor was also seen in Rab8 immunoprecipitates co-expressing optineurin or E50K mutant (Figure 11A). Quantitative analysis of the western blot showed that the amount of TfR was 96% more in Rab8 immunoprecipitate from the E50K expressing cells compared to wild type optineurin expressing cells. In accordance with these observations, the E50K mutant showed better colocalization with Q67L-Rab8 compared to wild type optineurin (correlation coefficient, E50K mutant 0.63 ± 0.12, wild type optineurin 0.28 ± 0.04; p < 0.01) (Figure 11B). We then examined the distribution of TfR in cells co-expressing E50K and Q67L-Rab8. Both the TfR and Rab8 (Q67L) were found together in the same vesicular structures as that of E50K but to a lesser extent in cells containing wild type optineurin (Figure 11B). Quantitative analysis of pair wise colocalization was carried out by calculating correlation coefficients (Table 2). This analysis suggests that Rab8 shows better colocalization with TfR in the E50K-expressing cells compared to wild type optineurin expressing cells. These observations provide support to our results showing that compared to wild type optineurin, the E50K mutant shows stronger interaction with Rab8 and TfR. Taken together these results suggest that optineurin associates with Rab8 and TfR through its different domains to form a trimolecular complex.

**Table 2:** Correlation coefficients for pairwise colocalization

Cells expressing	Colocalization/Proteins compared	Correlation coefficient
Wild-type optineurin and Q67L Rab8	Optineurin and Q67L-Rab8	0.29 ± 0.02*
	Q67L-Rab8 and TfR	0.42 ± 0.09**
	Optineurin and TfR	0.28 ± 0.03***

E50K mutant and Q67L-Rab8	E50K and Q67L-Rab8	0.63 ± 0.12*
	Q67L-Rab8 and TfR	0.58 ± 0.06**
	E50K and TfR	0.58 ± 0.07***

*p < 0.01; **p < 0.05; ***p < 0.01

In order to ascertain if the E50K mutant affects Rab8 function, we examined the dynamics of Rab8 vesicles in the presence of E50K mutant and wild type optineurin by time-lapse video microscopy. HeLa cells were cotransfected with GFP-Rab8 and HA-optineurin or E50K mutant or GFP-Rab8 alone. The movement of Rab8 vesicles was then monitored by acquiring a series of time-lapse images. Rab8 formed mostly elongated tubular structures with many smaller, rapidly moving vesicles. These Rab8 vesicles moved at an average velocity of 6.84 ± 1.38 μm/min. Co-expression of wild type optineurin only slightly reduced the velocity of Rab8 vesicles (5.83 ± 0.408 μm/min). In the cells co-expressing E50K, Rab8 formed conspicuous vesicles whose velocity was decreased to 2.64 ± 0.25 μm/min (P < 0.001) (Figure 11C). These results suggest that the E50K mutant optineurin affects trafficking of Rab8.

### The UBD is required for induction of cell death by the E50K mutant in RGC5 cells

**Figure 12:**
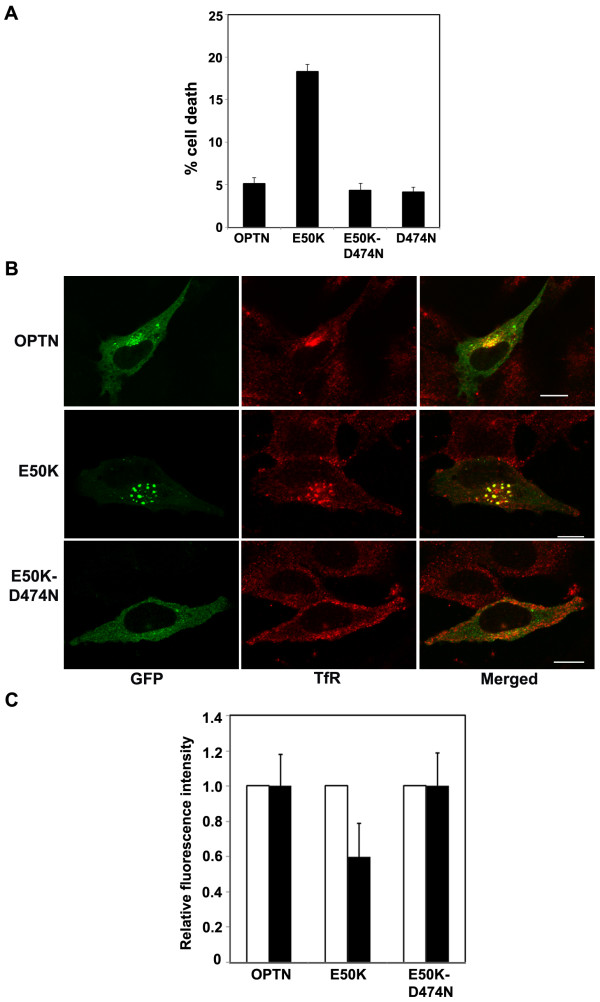
**The induction of cell death in RGC-5 cells by E50K mutant requires the UBD**. (A) RGC-5 cells were transfected with wild type optineurin or E50K mutant or their UBD mutants and percentage of cells undergoing cell death were quantified in transfected and untransfected cells. Data represent mean ± SD of 3 independent experiments. (B) E50K mutant colocalizes with TfR in RGC5 cells. GFP-tagged optineurin or its mutants were transfected in RGC-5 cells and stained for TfR. E50K mutant optineurin forms large vesicular structures that co-localize strongly with TfR indicated by yellow colour in the merged image. (C). E50K mutant causes reduced uptake of transferrin in RGC-5 cells. RGC-5 cells were transfected with GFP- tagged wildtype optineurin or its mutants and incubated with Alexa-546 conjugated transferrin for 20 min. Cells were then fixed and observed with a confocal microscope. The uptake of labelled transferrin was quantitated by measuring the fluorescence intensity in expressing (filled bar) and non-expressing (empty bar) cells. Bar; 10 μm.

Defective vision in glaucoma occurs due to apoptotic death of RGCs in the optic nerve head [35]. We have shown earlier that the E50K mutant of optineurin selectively induces the death of RGCs, and not of other cell lines tested [36], indicating that this mutation causes glaucoma by directly inducing the death of RGCs. To explore the role of the UBD of E50K in inducing death of RGCs, we studied the effect of the E50K-D474N double mutant on RGC5 cells, and found that it did not induce cell death (Figure 12A). In RGC5 cells also, the E50K mutant showed strong colocalization with TfR whereas the E50K-D474N mutant showed much less colocalization (correlation coefficient, E50K mutant 0.59 ± 0.09, double mutant 0.12 ± 0.09, wild type optineurin, 0.41 ± 0.11; p < 0.05) (Figure 12B). Expression of the E50K mutant in RGC5 cells reduced the uptake of transferrin whereas the double mutant did not (Figure 12C). These results show that the function of UBD is required for the retinal ganglion cell death induced by the E50K mutant of optineurin.

## Discussion

Optineurin gene is mutated in certain forms of glaucoma. The E50K mutation is the most common and severe disease causing mutation [11,38]. Nevertheless, the nature of functional alterations caused by mutations in optineurin and their probable role in etiopathogensis of glaucoma are unclear. Here we have investigated the role of optineurin in endocytic trafficking of TfR and how the E50K mutant affects this trafficking. Our results show that optineurin regulates trafficking of TfR to the juxtanuclear region and at least a fraction of endogenous as well as overexpressed optineurin is localized to the RE. This localization of optineurin to the RE is dependent on a functional UBD. The results of several independent experiments suggest that the E50K mutant causes impaired trafficking at the RE; these include: (a) E50K forms larger, TfR-containing vesicles (RE) than those formed by normal optineurin, (b) E50K vesicles show slower velocity and pause more frequently than those formed by normal optineurin, (c) the expression of E50K mutant (but not normal optineurin) reduces uptake of labeled transferrin by the cells, and (d) the expression of E50K reduces velocity of Rab8 vesicles.

A recent study has shown the presence of a novel UBD in optineurin through which it binds Lys63 linked poly-ubiquitin chains [3]. The ubiquitin binding-deficient mutants of optineurin and its E50K mutant failed to localize to the RE and did not form complex with TfR. These results suggest that the function of UBD is required for the localization of optineurin to the RE. The function of UBD is also needed for trafficking of transferrin to the juxtranuclear region because shRNA resistant D474N mutant could not rescue the effect of optineurin knockdown. Thus we have identified novel functions of the UBD of optineurin. Since optineurin interacts with TfR through UBD, it is likely that this interaction plays a role in the localization of optineurin to the RE.

Ubiquitination plays a key role in various aspects of vesicular trafficking, including internalization and the endocytosis of many cell surface receptors [39,40]. Ubiquitination of endocytosed receptors usually serves as a targeting signal for lysosome for their eventual degradation. The role of ubiquitination in endocytic recycling is uncertain. Our results indicate that TfR that is complexed with optineurin and the E50K mutant is ubiquitinated. It has been observed that TfR when tagged to ubiquitin is targeted to lysosomes [41]. Our observations provide first indication of the ubiquitination of TfR at the RE. However, this ubiquitination of TfR is likely to be very transient. Recently it has been shown that optineurin enhances ubiquitination of Tax1 protein of HTLV-1 raising the possibility that optineurin may be involved in regulating ubiquitination [42]. It is plausible that the overexperssed wild-type optineurin and its E50K mutant in particular either enhance this ubiquitination, or by their stronger association/affinity for ubiquitinated TfR, might prevent it's deubiquitination. However, the role of ubiquitination of TfR in its recycling needs further investigation. It is probable that the ubiquitination of TfR may be involved in the localization of optineurin to the RE.

Various studies have shown a role for Rab8 in trafficking at the recycling endosomes [20,21,23]. Interestingly, cells depleted of Rab8 are also defective in trafficking of TfR and exhibit a similar phenotype as that of optineurin depleted cells (this paper) implicating that both Rab8 and optineurin may regulate a common pathway in the trafficking of TfR [21]. Since optineurin interacts directly with Rab8, we suggest that optineurin, Rab8 and TfR form a trimolecular complex and recruit additional factors to facilitate transferrin/TfR trafficking to the RE. Stronger association and colocalization of Rab8 and TfR in presence of the E50K mutant suggest that the E50K mutant affects Rab8 mediated TfR trafficking. This suggestion is supported by the observation that the E50K mutant reduces the velocity of Rab8 vesicles.

Yue and colleagues have shown that in two ocular cell lines most of the endogenous optineurin is not present in the Golgi and overexpessed optineurin forms foci which co-localize with markers of the recycling endosomes [15]. The E50K mutant forms larger and more foci, and induces more cell death in retinal pigmented epithelial cells. It was speculated that the Golgi breakdown and/or defect in vesicle trafficking may be involved in the induction of cell death by the E50K mutant. Whether defective trafficking caused by the E50K mutant contributes to the Golgi breakdown is yet to be investigated.

E50K is a dominantly inherited mutation which shows strong association with glaucoma phenotype in a large family, suggesting therefore that it is a disease causing mutation [11]. The E50K mutant but not wild type optineurin causes death of RGC, indicating thereby that this mutation causes glaucoma by directly inducing death of RGC [36]. Since optineurin does not have any enzymatic activity it is likely that the altered interactions (direct or indirect) of the E50K mutant with cellular proteins might cause functional defects leading to RGC death. The E50K mutant shows enhanced interaction with a protein kinase, TBK1, although functional significance of this interaction is not known [43]. Here, we have shown that the E50K mutant causes impaired endocytic recycling of TfR, and altered interactions of this mutant with Rab8 and TfR possibly contribute to impaired trafficking. Impaired trafficking by the E50K mutant is likely to affect cellular homeostasis because constant recycling of receptors between the cell membrane and RE is vital for maintaining homeostasis of membrane components and nutrients, such as iron. Endocytic recycling also plays an important role in cellular signal transduction by many cell surface receptors [44,45]. Requirement of the UBD for impaired trafficking as well as induction of cell death in RGC by the E50K mutant indicates that the impaired trafficking caused by E50K might contribute to death of RGC, possibly by disrupting cellular homeostasis or signal transduction.

Patients with mutations in optineurin, such as E50K, have glaucoma and are not affected in other tissues although optineurin is expressed ubiquitously. It is likely that mutations in optineurin affect a function that is critical for the survival of neuronal cells. Some studies have shown blockade of axonal transport in glaucomatous conditions [46-48]. Axonal transport, especially of neurotrophins, is essential for the survival of neuronal cells including RGCs. Since E50K mutant impairs endocytic recycling, it is likely that this mutant causes defective axonal trafficking. The higher levels of optineurin in RGCs compared to brain might contribute to increased RGC death resulting in glaucoma with relatively little or no neuronal cell death elsewhere.

## Conclusions

Our results show that optineurin is required for trafficking of transferrin receptor to the juxtanuclear region. Optineurin is localized to the RE through UBD, which is also required for trafficking of TfR to the juxtanuclear region. Dynamic interactions of optineurin with Rab8 and TfR are likely to contribute to the endocytic recycling of TfR. A disease causing mutation, E50K, impairs endocytic recycling of TfR, possibly due to altered interactions with Rab8 and TfR. These results also have implications for the pathogenesis of glaucoma caused by the E50K mutation.

## Methods

### cDNA constructs and reagents

Plasmid vectors for expressing human optineurin and its mutants (E50K) with HA tag have been described [36]. These were cloned in pEGFP-C3 (Clonetech) to produce GFP- tag. Optineurin and E50K mutant without tag were produced by cloning the required cDNA in pcDNA3 plasmid. Human Rab7 and Rab11 were PCR amplified from A549 cell RNA and cloned in pEGFP-C3. Human ubiquitin cDNA was amplified by PCR and cloned in pGEX4T3 vector. Point mutations in optineurin and Rab8 were created by a PCR based site directed mutagenesis strategy. A mutant of optineurin resistant to degradation by shOPTN2 was generated by mutating four nucleotides in the region targeted for degradation by shOPTN2 without changing the amino acid sequence of the optineurin protein.

Mouse monoclonal anti-ubiquitin was from Calbiochem, rabbit polyclonal anti-optineurin was from Abcam, mouse monoclonal anti-transferrin receptor was from Zymed. mouse monoclonal anti-HA was from Roche Applied Biosystems. Rabbit polyclonal anti-HA was from SantaCruz, Alexa 546-conjugated transferrin was from Molecular Probes.

### Cell Culture and transfections

Retinal ganglion cell line RGC-5 has been described previously [49,50]. HeLa and RGC-5 cells were maintained at 37°C in a CO_2 _incubator as described [36]. Transfections were done using Lipofectamine Plus™ reagent (Invitrogen Life Technologies, Inc.) according to the manufacturer's instructions.

### Indirect immunofluorescence and confocal microscopy

For immunofluorescence, cells grown on coverslips were transfected with required plasmids, fixed and stained with appropriate antibodies, as described previously [51]. For analysis of co-localization cells were observed using a LSM 510 Meta or LSM 510 NLO confocal microscopes (Carl Zeiss Microimaging, Jena). For imaging GFP and cy3, a 488 nm argon laser and 543 nm or 561 nm DPSS laser was used. Serial optical sections in the *Z*-axis of the cells were collected at 0.33 μm intervals with a 63 × oil immersion objective lens (NA 1.4). Generally 2 serial optic sections were projected and colocalization was observed using LSM 510 (version 3.2) software. Quantitative analysis of colocalization was carried out by calculating Pearson's correlation coefficients using LSM 510 software. Sizes of about 120 vesicles from 10 different cells expressing wild type and mutant optineurin were estimated using LSM 510 (version 3.2) software. Images were further processed with Adobe Photoshop software. The number of vesicles (larger than 0.5 μm and 0.8 μm) in wild-type and mutant optineurin expressing cells was computed in at least 100 cells using Imaris software (Bitplane scientific solutions).

### Generation of adenoviral vectors

Endogenous optineurin was down-regulated by using adenoviral vectors expressing shRNAs as described previously using pAdEASY system [4,52,53].

Adenoviral vectors for expressing optineurin and its E50K mutant were prepared as described using pAdEASY system [4,53].

### Immunoprecipitation and GST pull down

Immunoprecipitations were carried as essentially as described [51]. The proteins were eluted by boiling in 3 × SDS sample buffer and resolved by SDS-PAGE. The proteins were transferred to nitrocellulose membrane for western blot analysis as described [54].

For GST pull down assays, GST, GST-ubiquitin were expressed in *E. coli *and were conjugated to Sepharose beads as described [55]. GST- or GST-ubiquitin beads were incubated 6-8 hours with lysates of HeLa cells transiently transfected with indicated plasmids. Bound proteins were eluted by boiling in 3 × SDS sample buffer and subjected to immunoblotting.

### Time-lapse microscopy

HeLa cells were plated on chambered coverglass (LabTek) and transfected with appropriate GFP-tagged constructs. Cells expressing GFP tagged constructs were observed with a Zeiss LSM 5 Live confocal microscope. GFP was excited by using a 488 nm diode laser and images were acquired at an interval of 12 secs or 5 secs using a 63 × oil or 100× oil immersion objectives (NA 1.4). During the imaging cells were maintained at 37°C and 5% CO_2_. The movement of the vesicles were tracked by using Metamorph software (Universal imaging). For calculating the velocities, 200 vesicles (~4800 measurements) in 12 E50K expressing cells and 160 vesicles (~2800 measurements) in 11 OPTN expressing cells were tracked. For calculating pausing, the total amount of time a vesicle remains stationary was determined and is expressed as a percentage [56]. A vesicle was considered to be stationary or paused, if the displacement is 1 pixel or less for at least 3 consecutive time points.

### Transferrin Uptake

After 24 hrs of transfections, HeLa cells grown on coverslips were washed and pre-incubated with serum free DMEM for 2 hrs. Cells were then incubated with 10 μg/ml of Alexa594 conjugated transferrin (Molecular Probes) in serum-free medium for 1 hr at 4°C. Cells were then shifted to 37°C for 25 min to label recycling endosomes, washed with PBS twice and fixed in 3.7% formaldehyde. For quantitative analysis, the fluorescence intensity of internalized transferrin was measured using ImageJ software (n = 180 cells for E50K, n = 160 cells for wild type optineurin). The fluorescence intensities of the transfected cells were normalized with non-expressing cells.

### Frap

HeLa cells grown in chambered coverglass were transfected with required plasmids. Photobleaching experiments were carried out using LSM510 NLO microscope. A 63 × water immersion objective was used for imaging. For bleaching, cells transfected with 250 ng of each EGFP-containing construct were excited with a 488 nm laser. Bleaching was carried out by selecting circular regions of interest of diameter 2 μm, using 100% of laser power with the pinhole kept at 2 airy units. To determine the rate of fluorescence recovery, fluorescence in these regions was measured over time by acquiring images at low laser power (0.6%) every 400 msecs for 3 min, and then normalised with respect to corresponding total cellular fluorescence at each individual time point to correct for bleaching during low power laser excitation.

### Cell Death Assays

Quantitative analysis of dead or apoptotic cells was carried out as described [36,57].

### Statistical analysis

Graphs represent average ± SD values. Statistical differences were calculated using Student's T-test. When significant differences were observed, P values for pair wise comparisons were calculated by using two-tailed T-test. P values less than 0.05 were considered significant.

## Abbreviations

TfR: Transferrin receptor; UBD: Ubiquitin binding domain; RE: Recycling endosome; shRNA: short hairpin RNA; RGC: Retinal ganglion cell.

## Authors' contributions

AN performed most of the experiments, participated in design of experiments, interpretation of data and writing of the manuscript. MLC, NJ, VR and NR performed some of the experiments. GS conceived the project, designed the experiments, interpreted the data and wrote the manuscript. DB is project coordinator. All authors have read and approved the final manuscript.

## Supplementary Material

Additional file 1 **Dynamics of vesicles formed by GFP-optineurin**. HeLa cell transfected with GFP-OPTN was imaged by time lapse video microscope. Images were acquired every 12 sec for 15 min and played at 5 frames per second. Movie shows the movement of vesicles. Bar, 5 μm. Click here for file

Additional file 2 **Dynamics of vesicles formed by GFP-E50K**. Images were acquired every 12 sec for 15 min and played at 5 frames per second. Movie shows the movement of vesicles with occasional fusion of smaller vesicles with larger vesicles in the perinuclear region. Bar, 5 μm. Click here for file
